# Giant Synovial Cyst of Thigh: A Rare Entity

**DOI:** 10.1155/2013/967215

**Published:** 2013-05-16

**Authors:** Kushagra Sinha, Rajesh Maheshwari, Atul Agrawal

**Affiliations:** Department of Orthopaedics, Himalayan Institute of Medical Sciences, Dehradun, Uttarakhand 248140, India

## Abstract

Synovial cyst occurs secondary to traumatic, degenerative, or inflammatory conditions. Synovial cysts represent abnormal distension of bursae, which communicate with the joint. Giant synovial cysts are typically due to rheumatoid arthritis, other causes being trauma and synovial pseudoarthrosis. A 33-year-old male presented to an outpatient clinic with a massive swelling on his posterolateral aspect of right thigh extending from upper one-third to the knee joint which had been increasing in size over the past six months. This was associated with dull aching pain. All laboratory investigations were within normal parameters. Even FNAC was inconclusive. With time, swelling was increasing in size. Ultrasound revealed the cystic nature of swelling. MRI showed large cystic lesion 24 × 10 × 12 cm in posterolateral aspect of thigh extending up to knee joint. Following the MRI, an excision was planned. After excision, histological examination confirmed the synovial nature of the cyst, which had a collagenous wall and dense chronic inflammatory cells. As the disease is extremely rare and asymptomatic, precise diagnosis is difficult and often delayed. We consider that open surgical excision should be reserved for cases of large synovial cysts because it can provide a complete resection of the lesion and minimize the risk of recurrence.

## 1. Case Report

Synovial cyst occurs secondary to traumatic, degenerative, or inflammatory conditions. Synovial cysts represent abnormal distension of bursae, which communicate with the joint [1]. The popliteal region is the commonest site of synovial cysts [2]. Giant synovial cysts are typically due to rheumatoid arthritis, other causes being trauma and synovial pseudoarthrosis [3].

## 2. Clinical Presentation

 A 33-year-old male presented to an outpatient clinic with a massive swelling on his posterolateral aspect of right thigh extending from upper one-third to the knee joint which had been increasing in size over the past six months. This was associated with dull aching pain. The patient also felt the mass to be aesthetically displeasing.

 Pain was aggravated by movement and alleviated to some extent by rest. There was no history of trauma, no history of any joint pain, and no personal and family history of gout, rheumatoid disease, or other arthritis.

**Figure 1:**
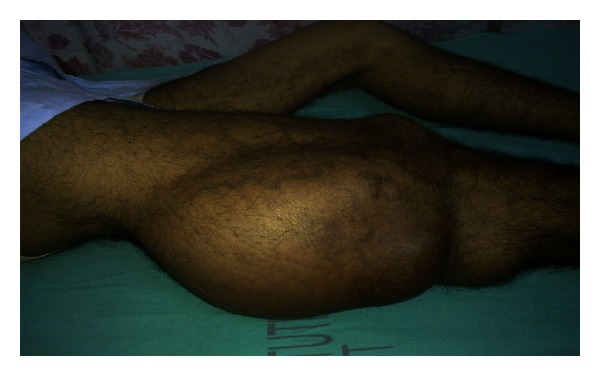
Clinical photograph showing cystic mass extending from knee to upper one-third thigh on posterolateral aspect.

Physical examination revealed 30 × 20 cm swelling, nontender, and the mass was cystic in consistency and transilluminated with well-defined margin (Figure 1). Flexion at the knee joint was restricted due to the size of the swelling and distal neurovascular status was intact. However, clinically, the swelling was not communicating to the knee joint. 

 Laboratory examination revealed haemoglobin of 14.4 gm%, total leukocyte count of 8500/mm^3^, differential leukocyte count of n_61_l_34_e_03 _m_02_, erythrocyte sedimentation rateof 25 mm/hr, total protein of 4.4 g/dL, albumin of 2.1 g/dL, blood urea nitrogen of 13 mg/dL, and random blood sugar of 133 mg/dL. Aspiration produced rusty coloured fluid. Synovial fluid protein and glucose were 5000 mg/dL, and 82 mg/dL respectively, rheumatoid factor was negative, C-reactive protein was normal, and serum uric acid was 4.2 mg/dL. Bacteriological cultures failed to grow any organisms. Cytology showed acute inflammatory pathology.

**Figure 2:**
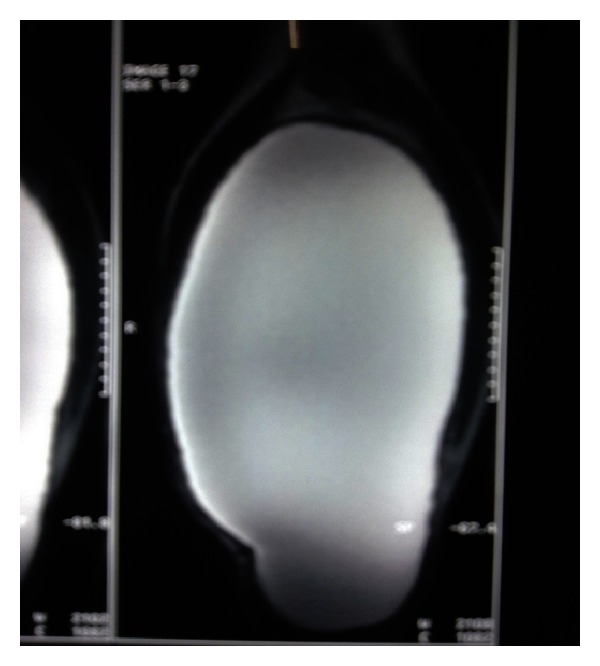
MRI right lower limb showing large cystic mass.

An ultrasound scan of the mass revealed the cystic nature of swelling. An MRI of the right lower limb was obtained which revealed a large cystic lesion 24 × 10 × 12 cm in posterolateral aspect of thigh extending up to knee joint. The lesion was closely related to the biceps femoris and hamstring muscle (Figure 2). Following the MRI, an excision was planned.

Our case was a large extra articular synovial cyst and this was the reason we believed that arthroscopic intervention cannot provide a complete resection of the cyst. In such cases, the possibility of leaving even a small piece of wall lining poses a high potential risk of recurrence. Therefore, an open surgical procedure was necessary.

**Figure 3:**
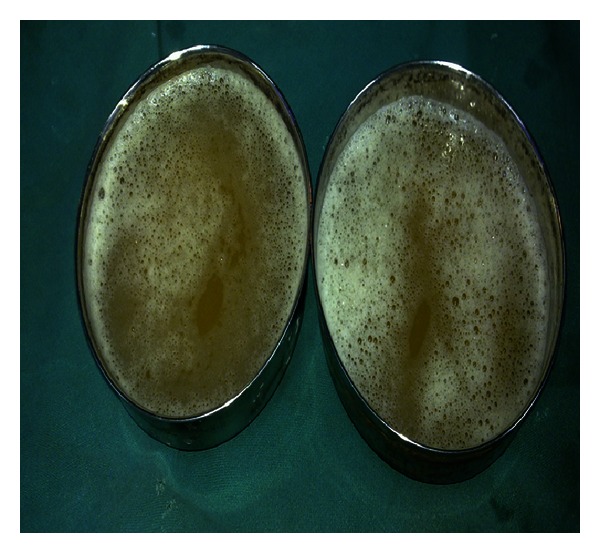
Serous fluid aspirated from the cyst.

As the skin over the swelling was so tense, aspiration was done before the surgical incision, and approximately 500 mL of fluid was aspirated and was found to contain thin, serous material (Figure 3).

**Figure 4:**
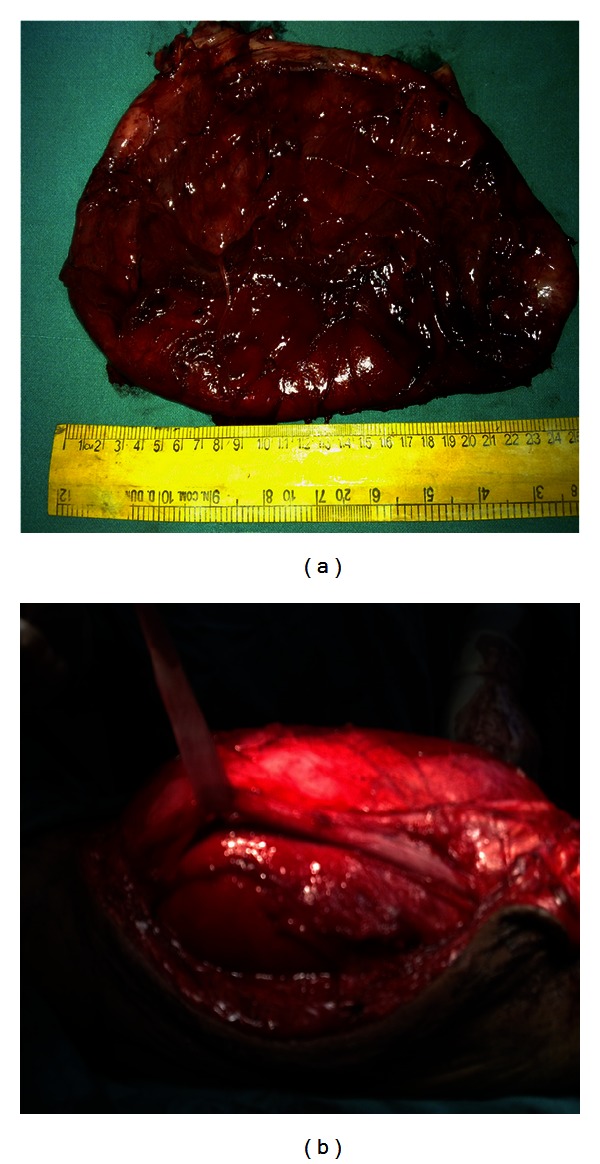
(a) shows size of a synovial cyst excised measuring 24 × 13 cm. (b) shows sciatic nerve separated from posterior wall of the cyst.

 Intraoperatively, the cyst measuring 24 × 13 cm was identified, which was adherent to the sciatic nerve posteriorly and popliteal vessels were displaced anterolaterally. Cyst was originating from the surface of hamstring and biceps femoris tendon sheath and adjacent posterior surface of femur. Cyst was extending proximally in thigh and no communication was found between the joint space and the cyst. The cyst was removed totally and the base of the cyst was cauterized (Figures 4(a) and 4(b)). The base was on the surface of tendon sheath and adjacent to the lower third posterior surface of right femur. 

**Figure 5:**
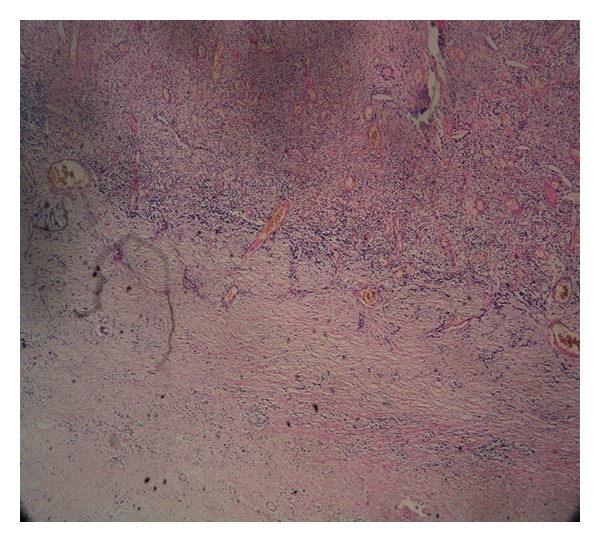
Histopathological slide showing collagenous wall and dense chronic inflammatory cells.

Histopathological examination confirmed the synovial nature of the cyst, which had a collagenous wall and dense chronic inflammatory cells (Figure 5).

Postoperatively, the patient was pain free and showed almost normal strength and sensation in the right thigh. The patient had uneventful recovery with complete resolution of all symptoms. No recurrence has been observed for three years.

## 3. Discussion 

 Synovial cysts can originate from joints, bursae, or tendon sheaths. It is difficult to discriminate between cysts from bursae and those from tendon sheaths, because both share a close anatomic relationship and a similar histological appearance. Synovial cyst constitutes para-articular fluid collection lined by synovial membrane, which may or may not communicate with the adjacent joint. Ganglion cysts may have a definable connection to the joint which seems, however, to be the most important mechanism for the development of ganglion cysts. The degree of intra-articular pressure is an important factor as well, which causes the enlargement of the ganglion cyst. Furthermore, a valve mechanism supports the development of these cysts [4, 5]. Typical aetiology factors include rheumatoid arthritis, seronegative spondyloarthropathies, osteoarthritis and crystal deposition diseases, trauma, or tumors [6, 7]. 

For any of these reasons, a shift of synovial fluid from regions of high to low pressure causing a progressive mobilization of the synovial tissue and its eventual outgrowth outside the capsule. Once this phenomenon occurs, the liquid may organize or move freely from the joint to the cyst through a channel with permeability that can be permanent, episodic, or short lasting. The displacement of fluid in one direction or another can increase or decrease in the size of the cavity [8, 9].

 Synovial cysts may cause pain or limitation of joint mobility. Uncommonly, they may cause compression of the neighbouring neurovascular structures. Van Mourik et al. reported a case of synovial cyst of hip joint presenting as deep venous thrombosis, secondary to compression of the femoral/iliac vein [10]. Acute rupture of the cysts may occur infrequently which may dissect into adjacent soft tissues. Secondary infection of the cysts may lead to abscess formation. Plain radiographs and ultrasound imaging have been used in evaluation of synovial cysts. However, MRI is considered the best imaging modality for optimal delineation of the extent of the vital structures and extent of the cyst. Aetiology of the giant synovial cyst could not be diagnosed. However, it is very rare for a synovial cyst to reach a size of 24 × 13 cm and we have not found any case reports similar to this. 

There is various treatment modalities available in form of aspiration and steroid administration, arthroscopic removal, and surgical excision described in the literature. Ultrasound-guided aspiration of symptomatic ganglion cysts is a valuable therapeutic modality because incomplete aspiration often results in cyst recurrence. Ultrasound guidance is especially useful for nonpalpable cysts, those near neurovascular bundles, and those with loculations and internal septations [11]. The drawback of this technique is that the mechanism that gives rise to the lesion may still be patent and, as a result, the cyst can replenish synovial fluid in time [8].

 Arthroscopic treatment is very useful when there is intra-articular communication and associated joint disorder which lead to the concept that these lesions should also be healed to obliterate these cysts and prevent their recurrence [12]. However, the surgeon should be highly skilled in the arthroscopic procedures. 

 Surgical excision is the definitive treatment but procedures have been tested including needle aspiration of intracystic content and administration of fibrin-based substances that affect the final sealing of the walls [13].

## 4. Conclusion

Extra-articular giant synovial cysts may enlarge considerably to manifest as mass lesions at locations away from the joint and may rupture leading to compartment syndrome, infection, or compress the neurovascular structures. As the disease is extremely rare and asymptomatic, precise diagnosis is difficult and often delayed. More attentions should be paid because of its severe complications.

We consider that open surgical excision should be reserved for cases of large synovial cysts because it can provide a complete resection of the lesion and minimize the risk of recurrence. On the other hand, arthroscopic treatment is more suitable for small lesions that lay strictly within the synovium. 

## References

[1] Perri JA, Rodnan GP, Mankin HJ (1968). Giant synovial cysts of the calf in patients with rheumatoid arthritis. *Journal of Bone and Joint Surgery—Series A*.

[2] Pastershank SP, Mitchell DM (1977). Knee joint bursal abnormalities in rheumatoid arthritis. *Canadian Association of Radiologists Journal*.

[3] White TK, Incavo SJ, Moreland MS (1988). Giant synovial cyst of the hip joint. *Orthopaedic Review*.

[4] Lu KH (2003). Unusual solitary ganglion cysts of the anterior segment of the lateral meniscus. *Arthroscopy*.

[5] Shetty GM, Nha KW, Patil SP (2008). Ganglion cysts of the posterior cruciate ligament. *Knee*.

[6] Morris CS, Beltran JL (1990). Giant synovial cyst associated with a pseudarthrosis of a rib: MR appearance. *American Journal of Roentgenology*.

[7] Bystrom S, Adalberth G, Milbrink J (1995). Giant synovial cyst of the hip: an unusual presentation with compression of the femoral vessels. *Canadian Journal of Surgery*.

[8] Mine T, Ihara K, Kawamura H, Kuwabara Y (2010). Intra-articular synovial cyst of the knee joint: a case report. *Journal of Orthopaedic Surgery (Hong Kong)*.

[9] Tschirch FTC, Schmid MR, Pfirrmann CWA, Romero J, Hodler J, Zanetti M (2003). Prevalence and size of meniscal cysts, ganglionic cysts, synovial cysts of the popliteal space, fluid-filled bursae, and other fluid collections in asymptomatic knees on MR imaging. *American Journal of Roentgenology*.

[10] van Mourik JBA, Josaputra HA, Axler A (1988). Giant synovial cyst causing deep venous thrombosis: brief report. *Journal of Bone and Joint Surgery—Series B*.

[11] Saboeiro GR, Sofka CM (2008). Ultrasound-guided ganglion cyst aspiration. *HSS Journal*.

[12] Shetty GM, Wang JH, Ahn JH, Lee YS, Kim BH, Kim JG (2008). Giant synovial cyst of knee treated arthroscopically through a cystic portal. *Knee Surgery, Sports Traumatology, Arthroscopy*.

[13] Mifsut D, Llorente MJ, Sanchez F, Jerome D, McKendry RJ (2001). Unusual synovial cyst of the knee treated with fibrin sealant. *Journal of Rheumatology*.

